# Predictive value of TyG-BMI, CTI, and SII in identifying metabolic dysfunction-associated steatotic liver disease among patients with type 2 diabetes mellitus

**DOI:** 10.3389/fnut.2026.1808180

**Published:** 2026-05-08

**Authors:** Yue Chen, Yayu Lai, Qu Xiang, Li Fan, Xingyu Chen, Jin Gao, Qingfeng Cheng, Xu Luo

**Affiliations:** 1 Quality Control Department of Jiangbei Campus, The First Affiliated Hospital of Army Medical University (The 958th Hospital of the Chinese People's Liberation Army), Chongqing, China; 2 Department of Cardiology of Jiangbei Campus, The First Affiliated Hospital of Army Medical University (The 958th Hospital of the Chinese People's Liberation Army), Chongqing, China; 3 Infection Control Department of Jiangbei Campus, The First Affiliated Hospital of Army Medical University (The 958th Hospital of the Chinese People's Liberation Army), Chongqing, China; 4 Department of Endocrinology of Jiangbei Campus, The First Affiliated Hospital of Army Medical University (The 958th Hospital of the Chinese People's Liberation Army), Chongqing, China; 5 Department of Endocrinology, The First Affiliated Hospital of Chongqing Medical University, Chongqing, China; 6 The Office of Jiangbei Campus, The First Affiliated Hospital of Army Medical University (The 958th Hospital of the Chinese People's Liberation Army), Chongqing, China

**Keywords:** C-reactive protein–triglyceride–glucose index, metabolic dysfunction-associated steatotic liver disease, predictive model, systemic immune-inflammation index, triglyceride-glucose body mass index, type 2 diabetes mellitus

## Abstract

**Background:**

Metabolic dysfunction–associated steatotic liver disease (MASLD) has become a major comorbidity in patients with type 2 diabetes mellitus (T2DM). This study aimed to evaluate the diagnostic performance of the triglyceride-glucose–body mass index (TyG-BMI), the C-reactive protein–triglyceride–glucose index (CTI), and the systemic immune-inflammation index (SII), individually and in combination, for identifying MASLD in patients with T2DM.

**Methods:**

This retrospective cross-sectional study enrolled 796 hospitalized patients with T2DM from January 2020 to January 2025. Sample size was determined based on available eligible cases during the study period. Data distribution was assessed using the Kolmogorov–Smirnov test. There were 280 MASLD patients and 516 non-MASLD patients. We collected anthropometric, biochemical, and inflammatory parameters. We performed correlation and multivariate logistic regression analyses to find independently associated factors. We evaluated diagnostic performance using receiver operating characteristic (ROC) curves, the area under the curve (AUC), and the Youden index. We also used net reclassification improvement (NRI) and integrated discrimination improvement (IDI) to assess predictive value. External validation was performed using an independent cohort of 394 patients. Key limitations include the retrospective design, potential misclassification due to ultrasound-based diagnosis, and residual confounding.

**Results:**

MASLD patients exhibited significantly higher BMI, triglycerides, low-density lipoprotein cholesterol (LDL-C), TyG-BMI, CTI, and SII levels than non-MASLD patients (all *p* < 0.05). TyG-BMI, CTI, and SII were independent MASLD predictors. The combined model (TyG-BMI + CTI + SII) had the highest discriminative power, outperforming each single indicator. Adding CTI and SII to TyG-BMI significantly improved classification (NRI = 0.312, IDI = 0.143, both *p* < 0.05). External validation indicated strong performance (AUC = 0.940, sensitivity = 84.6%, specificity = 90.1%). Machine learning validation using a random forest algorithm further indicated the high predictive accuracy of the model, achieving an AUC of 0.950 in the development cohort and maintaining a robust AUC of 0.885 in the external validation cohort.

**Conclusion:**

TyG-BMI, CTI, and SII are independently associated with MASLD in T2DM patients. Their combined use significantly improves early identification accuracy and could serve as a convenient, cost-effective screening tool in clinical practice.

## 1 Introduction

Metabolic dysfunction-associated steatotic liver disease (MASLD), formerly known as non-alcoholic fatty liver disease (NAFLD) or metabolic dysfunction-associated fatty liver disease (MAFLD), has emerged as a leading cause of chronic liver disease worldwide (1, 2). The transition from NAFLD to MASLD represents a paradigm shift. It emphasizes positive diagnostic criteria based on hepatic steatosis and specific cardiometabolic risk factors, providing a more accurate reflection of the disease’s metabolic basis (3, 4). Understanding these evolving definitions is crucial to appreciating current diagnostic challenges.

MASLD is characterized by hepatic steatosis with one or more cardiometabolic risk factors, such as obesity, insulin resistance, dyslipidemia, or hypertension (5). Among these, type 2 diabetes mellitus (T2DM) is recognized as the most significant driver of MASLD progression (6, 7). Epidemiological studies have shown that over 50–65% of T2DM patients have concomitant MASLD. The coexistence of these conditions markedly increases the risk of cirrhosis, hepatocellular carcinoma, and cardiovascular mortality (8, 9). Therefore, biomarkers reflecting both metabolic dysregulation and systemic inflammation may provide valuable insight into early disease detection and risk stratification. Liver biopsy, though the diagnostic gold standard, is invasive and unsuitable for large-scale screening (10). Imaging methods, such as ultrasound and MRI-based proton density fat fraction (MRI-PDFF), are noninvasive but costly and less accessible (11, 12). Reliable, readily available, and cost-effective biochemical markers are urgently needed to identify MASLD in T2DM patients.

Previous studies focused mainly on conventional metabolic indices. The triglyceride-glucose index (TyG) and its derivatives are reliable surrogates for insulin resistance and predictors of hepatic steatosis (13). Yet, MASLD pathogenesis includes both metabolic dysregulation and inflammatory changes. Inflammatory processes drive the progression from simple steatosis to steatohepatitis (14, 15). The C-reactive protein–triglyceride–glucose index (CTI) integrates metabolic and inflammatory components, reflecting their interaction at a systemic level (16). The systemic immune-inflammation index (SII), calculated from neutrophil, lymphocyte, and platelet counts, serves as a sensitive indicator of chronic inflammation (17). While each marker is individually linked to metabolic disorders, the combined predictive value of these markers for MASLD in diabetic patients remains unclear.

The study aimed to examine the association between TyG-BMI, CTI, and SII and MASLD in patients with T2DM. We also sought to build and externally validate a combined model to identify MASLD risk. We hypothesized that combining metabolic and inflammatory markers would improve predictive accuracy compared to using single indices. These results may provide a practical tool for early screening and clinical decision-making in the management of metabolic liver complications in diabetic populations.

## 2 Materials and methods

### 2.1 Study design and participants

This cross-sectional study retrospectively enrolled 796 adult inpatients diagnosed with type 2 diabetes mellitus (T2DM) at the 958th hospital of the Chinese People’s Liberation Army between January 2020 and January 2025. Inclusion criteria were: (1) diagnosis of T2DM according to the American Diabetes Association (ADA) 2025 criteria (18); and (2) availability of liver imaging or diagnostic records for MASLD evaluation. Exclusion criteria included: (1) viral hepatitis, autoimmune liver disease, or drug-induced liver injury; (2) excessive alcohol consumption (>30 g/day for men or >20 g/day for women); (3) malignancy or acute infection; and (4) missing laboratory data. The study was conducted in accordance with the Declaration of Helsinki and approved by the Ethics Committee of the 958th hospital of the Chinese People’s Liberation Army (Approval No: ER2025KY086). Due to the retrospective nature of the study and the use of previously collected anonymized clinical data without any patient contact, the ethics committee waived the requirement for informed consent. All patient identifiers were removed to ensure confidentiality, and the research project involved no personal privacy or commercial interests.

To evaluate the predictive models’ generalizability, an independent validation cohort was identified from the Department of Endocrinology, at the First Affiliated Hospital of Chongqing Medical University. This cohort covers the period from July 2020 to June 2025. The local Ethics Committee approved the study (Approval No. ZZ2025-551-01) in accordance with the Declaration of Helsinki. A total of 394 T2DM patients who met the development cohort’s inclusion and exclusion criteria were retrospectively enrolled.

### 2.2 Diagnostic criteria for MASLD and T2DM

MASLD diagnosis was based on hepatic steatosis confirmed by ultrasonography. It also required at least one metabolic risk factor, including overweight or central obesity, hyperglycemia, high blood pressure, raised plasma triglycerides, or reduced plasma high-density lipoprotein-cholesterol (3, 19).

T2DM was defined by fasting plasma glucose ≥7.0 mmol/L, 2-h postprandial glucose ≥11.1 mmol/L, HbA1c ≥ 6.5%, or in an individual with classic symptoms of hyperglycemia or hyperglycemic crisis, a random plasma glucose ≥11.1 mmol/L (18).

Patients were classified into MASLD and non-MASLD groups based on these criteria. The diagnosis recorded in the electronic medical records was confirmed by two hepatologists who independently reviewed the imaging reports and metabolic data.

All inclusion and exclusion criteria were predefined prior to data extraction and strictly applied to ensure consistency. Data collection followed standardized clinical and laboratory protocols across both centers to minimize measurement bias.

### 2.3 Data collection and laboratory measurements

Demographic, clinical, and laboratory data were extracted from electronic medical records. This included age, sex, diabetes duration, body mass index (BMI), blood pressure, smoking status, fasting plasma glucose (FPG), glycated hemoglobin (HbA1c), lipid profiles, high-sensitivity C-reactive protein (hs-CRP), and complete blood cell counts. All biochemical assays were conducted in the hospital’s central laboratory on fasting blood samples using standardized protocols.

### 2.4 Definition and calculation of metabolic and inflammatory indices

The three primary indices were calculated as follows (20–22):

$$\begin{array}{l} \mathrm{TyG}-\mathrm{BMI}=\mathrm{TyG}\times \mathrm{BMI},\text{where}\;\mathrm{TyG}=\\ \mathrm{Ln}\;[\text{fasting}\;\mathrm{TG}\;(\mathrm{mg}/\mathrm{dL})\times \text{fasting glucose}\;(\mathrm{mg}/\mathrm{dL})/2] \end{array}$$

$$\begin{array}{l} \mathrm{CTI}=0.412\times \mathrm{Ln}\;[\mathrm{hs}-\mathrm{CRP}\;(\mathrm{mg}/\mathrm{dL})]+\\ \mathrm{Ln}\;[\text{triglyceride}\;(\mathrm{mg}/\mathrm{dL})\times \text{fasting glucose}\;(\mathrm{mg}/\mathrm{dL})/2] \end{array}$$

SII=(Platelet count×Neutrophil count)/Lymphocyte count

To assess collinearity among TyG-BMI, CTI, and SII, variance inflation factors (VIF) were calculated; VIF < 5 indicated no significant multicollinearity. A correlation matrix of the three indices is provided in Supplementary Table 1.

### 2.5 Machine learning model construction

To explore non-linear relationships between metabolic–inflammatory indices and MASLD, several machine learning classifiers, including k-nearest neighbors, support vector machine, random forest (RF), gradient boosting machine, and XGBoost, were initially evaluated in the development cohort. The data were randomly split into a training-set and a test-set at ratio of 4:1. In the training stage, we performed 10-fold cross validation and repeated this process 10 times to find the optimal parameters of the models and to avoid overfitting. Specifically, for each of 10 repetitions, the training-set was divided into 10 equally sized subsamples, of which 9 were used to train a model and the 10th was used to evaluate its performance (23). After that, the test-set was used to assess the generalizability of models. Model performances were measured by recall, precision, specificity, AUC, accuracy, F1-score. The comparative performance of these algorithms is summarized in Supplementary Table 2. Among them, the RF model demonstrated the best overall performance and was therefore selected for further analysis.

The RF model was constructed using TyG-BMI, CTI, and SII as primary input features. The process of building models is the same as described above.

### 2.6 Statistical analysis

All statistical analyses were performed using SPSS version 26.0 and R software version 4.3.1. Continuous variables were expressed as mean ± standard deviation (SD) or median (interquartile range, IQR) according to distribution normality assessed by the Kolmogorov–Smirnov test and visual inspection of histograms. In contrast, categorical variables were expressed as counts (percentages). Group comparisons were conducted using the independent t-test or Mann–Whitney U test for continuous variables, and the *χ*^2^ test or Fisher’s exact test for categorical variables. Correlations among variables were evaluated using Pearson correlation coefficients. Due to the retrospective nature of the study, formal *a priori* sample size or power calculations were not performed. However, the relatively large sample size and external validation cohort enhance the robustness of the findings.

Variables with *p* < 0.10 in univariate analyses were included in multivariate logistic regression to identify independently associated factors. Calibration of the combined model was assessed using the Hosmer-Lemeshow goodness-of-fit test. The Brier score was calculated as an overall measure of prediction error. The diagnostic performance of TyG-BMI, CTI, and SII—individually and in combination—was assessed using receiver operating characteristic (ROC) analysis. The AUC, sensitivity, specificity, and Youden index were calculated to assess discrimination. The net reclassification improvement (NRI) and integrated discrimination improvement (IDI) quantified incremental predictive value. All statistical tests were two-tailed, and a *p* value < 0.05 was considered statistically significant. Confidence intervals were reported at the 95% level.

## 3 Results

### 3.1 Baseline characteristics of the study population

A total of 796 patients with T2DM were enrolled, including 280 (35.2%) patients diagnosed with MASLD. Table 1 summarizes the baseline characteristics of participants according to MASLD status. Patients with MASLD exhibited significantly higher BMI, diastolic blood pressure (DBP), triglyceride levels, LDL-C levels, and hs-CRP concentrations compared with the non-MASLD group (*p* < 0.05). In contrast, HDL-C levels were significantly lower in the MASLD group (*p* < 0.05). Other metabolic parameters, including age, sex distribution, diabetes duration, and glycemic indices (FPG, HbA1c), did not differ significantly between groups (*p* > 0.05).

**Table 1:** Baseline characteristics of T2DM patients with and without MASLD.

Variable	Total (*n* = 796)	Non-MASLD (*n* = 516)	MASLD (*n* = 280)	*p* value	SMD (95% CI)
Age (years)	68.00 (56.00, 75.00)	68.00 (57.00, 75.00)	66.00 (55.25, 74.00)	0.133	0.12 (−0.02 to 0.26)
Male, *n* (%)	436 (54.78)	273 (52.9)	163 (58.2)	0.151	0.11 (−0.03 to 0.25)
Diabetes duration (years)	11 (7.00, 18.00)	10 (6.25, 18.75)	12 (8.00, 18.00)	0.135	0.14 (−0.01 to 0.29)
BMI (kg/m^2^)	24.46 ± 4.03	23.45 ± 3.44	25.92 ± 2.81	<0.001	0.76 (0.62–0.90)
SBP (mmHg)	134.00 (122.25, 147.00)	133.00 (121.25, 146.00)	136.00 (124.00, 149.00)	0.052	0.20 (0.06–0.34)
DBP (mmHg)	80.00 (72.00, 89.00)	79.00 (71.00, 87.00)	83.00 (75.00, 92.00)	<0.001	0.42 (0.28–0.56)
Current Smoker, *n* (%)	399 (50.1)	261 (50.6)	138 (49.3)	0.727	−0.03 (−0.17 to 0.11)
FPG (mmol/L)	7.97 (6.41, 10.69)	7.72 (6.32, 10.44)	8.43 (6.63, 11.49)	0.088	0.21 (0.07–0.35)
HbA1c (%)	8.49 ± 2.43	8.48 ± 2.42	8.51 ± 2.45	0.872	0.01 (−0.13 to 0.15)
TG (mmol/L)	1.83 (1.23, 2.76)	1.57 (1.09, 2.20)	2.51 (1.70, 3.70)	<0.001	0.89 (0.74–1.04)
HDL-C (mmol/L)	1.17 ± 0.37	1.21 ± 0.36	1.09 ± 0.36	<0.001	−0.33 (−0.47 to −0.19)
LDL-C (mmol/L)	2.75 (1.99, 3.47)	2.58 (1.93, 3.40)	2.91 (2.13, 3.65)	<0.001	0.35 (0.21–0.49)
hs-CRP (mg/L)	2.44 (1.04, 5.00)	1.37 (0.73, 2.98)	4.88 (3.15, 7.72)	<0.001	1.12 (0.97–1.27)
Neutrophil (10^9^/L)	4.87 (3.61, 6.27)	4.45 (3.34, 5.93)	4.92 (3.65, 6.82)	0.115	0.17 (0.03–0.31)
Lymphocyte (10^9^/L)	1.52 ± 0.59	1.54 ± 0.58	1.48 ± 0.60	0.172	−0.10 (−0.24 to 0.04)
Platelet (10^9^/L)	186.28 (148.33, 226.80)	184.29 (136.67, 232.00)	189.83 (160.25, 219.67)	0.136	0.13 (−0.01 to 0.27)

The SMD (Standardized Mean Difference) is calculated by dividing the mean difference by the pooled standard deviation; for categorical variables (such as gender or smoking status), the SMD uses Cohen’s h.

Clinically, the higher BMI and TG levels in the MASLD group reflect the central role of obesity and dyslipidemia in MASLD pathogenesis; the lower HDL-C and elevated LDL-C indicate an atherogenic lipid profile; and the markedly elevated hs-CRP suggests subclinical inflammation commonly observed in MASLD. Other variables, including age, sex, diabetes duration, and glycemic indices (FPG, HbA1c), showed negligible effect sizes (all SMD < 0.25), indicating no clinically meaningful differences between groups.

### 3.2 Comparison of TyG-BMI, CTI, and SII between MASLD and non-MASLD groups

As shown in Table 2, all three indices differed significantly between the MASLD and non-MASLD groups (all *p* < 0.05). TyG-BMI and SII exhibited right-skewed distributions and were therefore summarized as median (interquartile range) and analyzed using nonparametric methods, whereas CTI followed an approximately normal distribution and was presented as mean ± standard deviation.

**Table 2:** Comparison of TyG-BMI, CTI, and SII between groups.

Variable	Non-MASLD (*n* = 516)	MASLD (*n* = 280)	*p* value
TyG-BMI	215.29 (194.44, 240.02)	258.85 (222.48, 288.09)	<0.001
CTI	9.42 ± 0.79	10.41 ± 0.72	<0.001
SII	544.47 (468.01, 629.54)	788.27 (679.77, 889.20)	<0.001

In terms of magnitude, TyG-BMI was markedly higher in the MASLD group (median: 258.85 vs. 215.29), representing an approximate 20.2% increase. Similarly, CTI levels were significantly elevated (10.41 ± 0.72 vs. 9.42 ± 0.79), corresponding to an approximate 10.5% increase. SII showed the most pronounced difference, increasing from 544.47 to 788.27, which represents an approximate 44.8% elevation in the MASLD group.

Collectively, these results indicate that higher levels of metabolic and inflammatory stress are strongly associated with MASLD in T2DM patients. To further illustrate the distributional differences, box plots of these indices have been added in Supplementary Figure 1.

### 3.3 Association between metabolic and inflammatory indices and MASLD

Correlation analyses demonstrated significant positive associations between TyG-BMI, CTI, SII, and the presence of MASLD (Table 3). These correlation analyses were unadjusted and reflect crude associations between each index and MASLD status. Among the three indices, CTI exhibited the strongest correlation with MASLD (*r* = 0.526, 95% CI 0.474–0.574, *p* < 0.05), followed by SII (*r* = 0.494, 95% CI 0.440–0.545, *p* < 0.05) and TyG-BMI (*r* = 0.434, 95% CI 0.376–0.489, *p* < 0.05). In terms of strength, these correlations can be considered moderate (*r* ≈ 0.4–0.5), indicating a meaningful but not exclusive contribution of these indices to MASLD status. These findings suggest that both metabolic dysregulation and systemic inflammation are closely associated with MASLD in patients with T2DM.

**Table 3:** Correlations between metabolic-inflammatory indices and MASLD.

Variable	Correlation coefficient (*r*)	95% CI	*p* value
TyG-BMI	0.434	0.376–0.489	<0.001
CTI	0.526	0.474–0.574	<0.001
SII	0.494	0.440–0.545	<0.001

To further account for potential confounding effects, multivariate logistic regression analyses were subsequently performed (Section 3.4), adjusting for key clinical covariates.

### 3.4 Factors associated with MASLD in patients with T2DM

Multivariate logistic regression analysis identified DBP, TyG-BMI, CTI, and SII as factors independently associated with MASLD after adjustment for potential confounders (Table 4). Covariates included in the model were selected based on clinical relevance and univariate screening (*p* < 0.10), including blood pressure and lipid profile variables. Composite indices (TyG-BMI, CTI, and SII) were retained to avoid redundancy and collinearity with their individual components.

**Table 4:** Multivariate logistic regression analysis for MASLD.

Variable	*β*	SE	*Wald χ^2^*	*p* value	OR (95% CI)
SBP (mmHg)	−0.004	0.007	0.444	0.505	0.996 (0.983–1.009)
DBP (mmHg)	0.031	0.011	8.498	0.004	1.032 (1.010–1.053)
HDL-C (mmol/L)	0.052	0.296	0.031	0.861	1.053 (0.590–1.880)
LDL-C (mmol/L)	0.117	0.084	1.949	0.665	1.042 (0.864–1.256)
TyG-BMI	0.016	0.003	28.719	<0.001	1.016 (1.010–1.022)
CTI	1.378	0.168	67.678	<0.001	3.968 (2.858–5.511)
SII	0.006	0.001	94.434	<0.001	1.006 (1.005–1.007)

Model adjusted for age, sex, diabetes duration, SBP, DBP, HDL-C, LDL-C, FPG, and HbA1c.

Specifically, TyG-BMI (OR = 1.016, 95% CI 1.011–1.022, *p* < 0.05), CTI (OR = 5.726, 95% CI 4.376–7.493, *p* < 0.05), and SII (OR = 1.006, 95% CI 1.005–1.008, *p* < 0.05) were significantly associated with increased odds of MASLD, suggesting that both metabolic and inflammatory disturbances contribute to disease risk. In contrast, HDL-C and systolic blood pressure (SBP) were not significantly associated with MASLD in the multivariate model (*p* > 0.05). Among the included indices, CTI demonstrated the largest effect size, suggesting that integrating inflammatory and metabolic components may better capture the underlying pathophysiological processes of MASLD.

### 3.5 Diagnostic performance of TyG-BMI, CTI, and SII for MASLD

**Figure 1:**
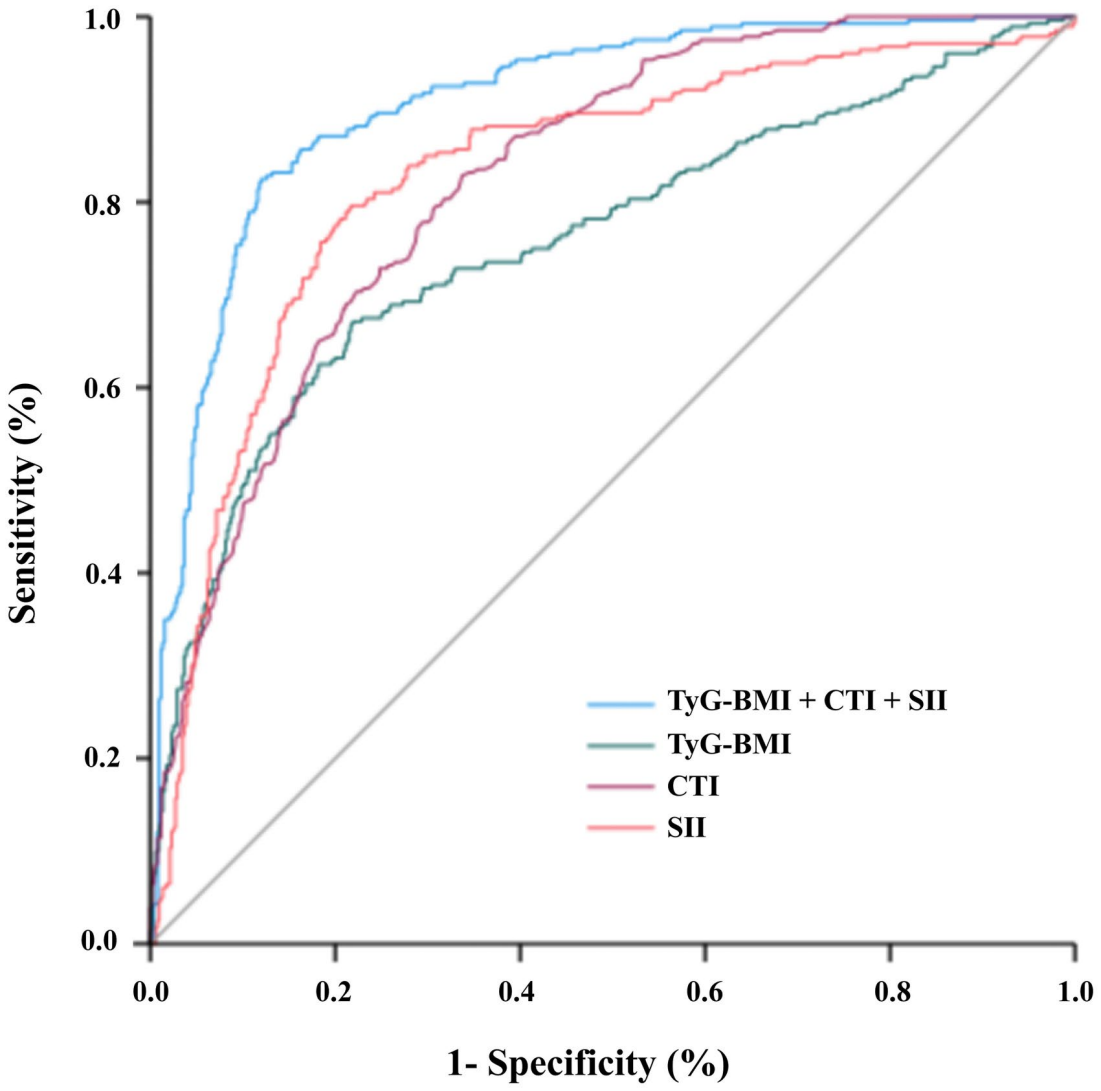
Receiver operating characteristic (ROC) curves for predicting MASLD in patients with T2DM using TyG-BMI, CTI, SII, and their combined model in the development cohort (*n* = 796). The area under the curve (AUC) for the combined model was 0.908 (95% CI: 0.887–0.929). Individual indices achieved the following AUCs: CTI, 0.823; SII, 0.829; TyG-BMI, 0.757. The dashed diagonal line indicates no discriminatory power (AUC = 0.5).

The ROC analysis results are presented in Figure 1 and Table 5. Among the individual indices, CTI and SII showed good discriminative power for MASLD (AUCs = 0.823 and 0.829, respectively), whereas TyG-BMI demonstrated moderate performance (AUC = 0.757). The combined model (TyG-BMI + CTI + SII) achieved the highest diagnostic accuracy. The AUC was 0.908 (95% CI: 0.887–0.929), with a sensitivity of 82.5%, specificity of 88.0%, and a Youden index of 0.705.

**Table 5:** Diagnostic performance of individual and combined indices for MASLD.

Model	AUC (95% CI)	Sensitivity (%)	Specificity (%)	Optimal cutoff	Youden’s Index
TyG-BMI	0.757 (0.719–0.794)	67.1	78.1	TyG-BMI > 213.40	0.452
CTI	0.823 (0.795–0.852)	82.9	66.3	CTI > 9.48	0.492
SII	0.829 (0.798–0.860)	75.7	81.6	SII > 571.23	0.579
TyG-BMI + CTI + SII	0.908 (0.887–0.929)	82.5	88.0	Predicted probability > 0.385	0.705

The optimal cutoff value for the combined model (based on the Youden index) was a predicted probability of 0.385. For clinical use, this means that a T2DM patient with a model-predicted probability >0.385 should be considered at high risk for MASLD. The sensitivity of 82.5% indicates that the model correctly identifies approximately 8 out of 10 MASLD patients, while the specificity of 88.0% means it correctly rules out nearly 9 out of 10 non-MASLD patients, making it suitable as a screening tool in high-risk T2DM populations.

To formally compare the discriminative performance between models, pairwise comparisons of AUCs were conducted using the DeLong test. The combined model demonstrated significantly higher AUC than TyG-BMI (ΔAUC = 0.1512, 95% CI: 0.117–0.185, *p* < 0.001), CTI (ΔAUC = 0.0844, 95% CI: 0.061–0.108, *p* < 0.001), and SII (ΔAUC = 0.0787, 95% CI: 0.054–0.103, *p* < 0.001). No significant difference was observed between CTI and SII (*p* = 0.782). Detailed results are presented in Supplementary Table 3.

The combined model showed good calibration (Hosmer-Lemeshow *χ*^2^ = 8.24, *p* = 0.410; Brier score = 0.142).

### 3.6 Incremental predictive value of combined models

The incremental predictive value analyses using NRI and IDI are summarized in Table 6. Adding CTI or SII individually to the TyG-BMI model significantly improved classification accuracy (NRI = 0.176 and 0.201, respectively; both *p* < 0.05). When both CTI and SII were incorporated simultaneously, the improvement was more pronounced (NRI = 0.312, IDI = 0.143; both *p* < 0.05), demonstrating a substantial enhancement in the model’s discriminative and reclassification performance. These findings confirm that the joint assessment of CTI and SII meaningfully refines MASLD risk prediction beyond the traditional TyG-BMI index.

**Table 6:** Incremental predictive value of combined models (vs.  TyG-BMI alone).

Model comparison	NRI (95% CI)	*p* value	IDI (95% CI)	*p* value
+ CTI	0.176 (0.092–0.260)	<0.001	0.071 (0.043–0.098)	<0.001
+ SII	0.201 (0.115–0.286)	<0.001	0.082 (0.051–0.114)	<0.001
+ CTI + SII	0.312 (0.220–0.404)	<0.001	0.143 (0.104–0.182)	<0.001

### 3.7 External validation of the prediction model

**Figure 2:**
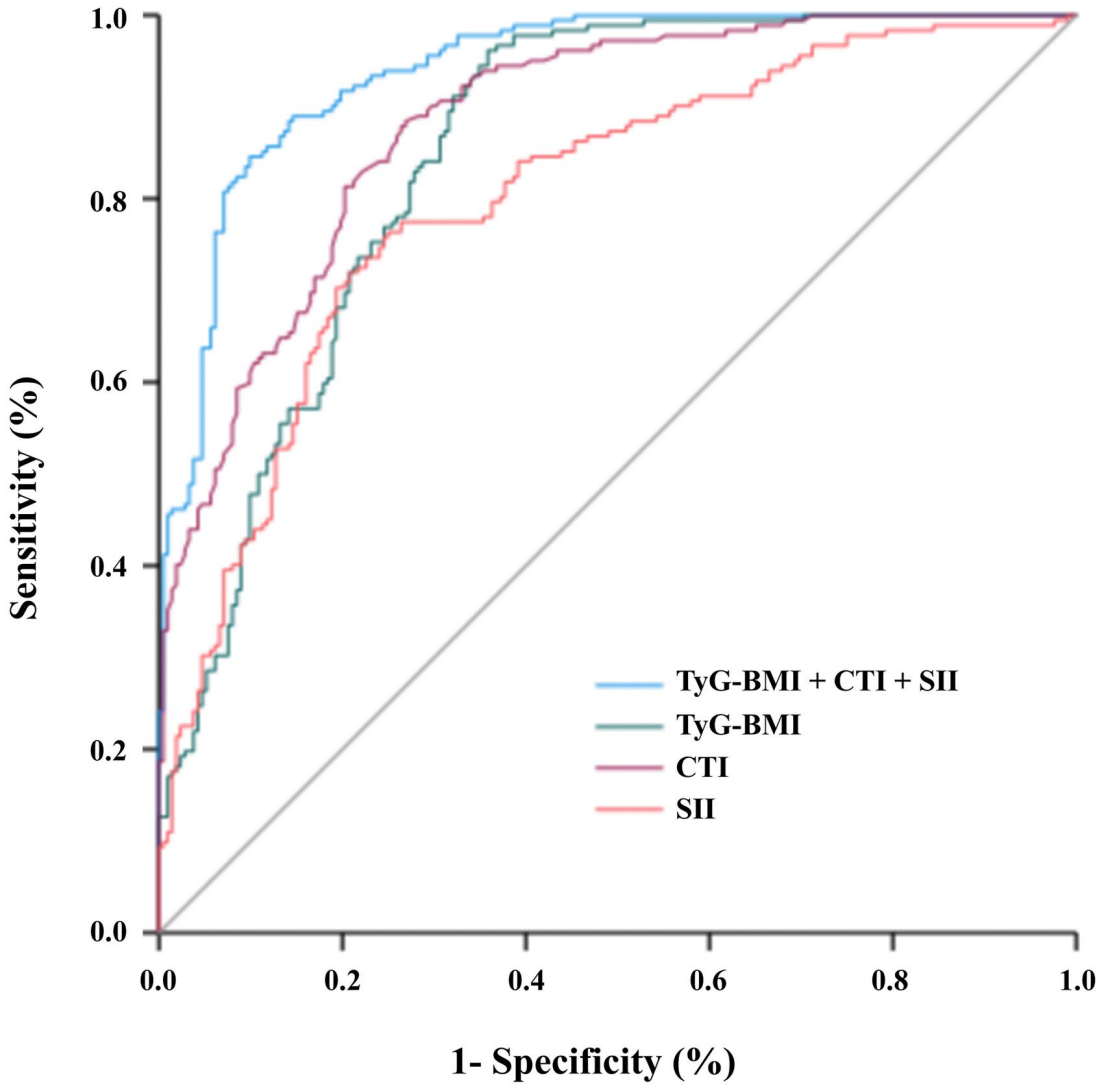
Receiver operating characteristic (ROC) curves of TyG-BMI, CTI, SII, and the combined model for MASLD prediction in the external validation cohort (*n* = 394). Receiver operating characteristic (ROC) curves show the performance of TyG-BMI, CTI, SII, and their combination. The combined model achieved an AUC of 0.940 (95% CI: 0.918–0.962), with 84.6% sensitivity and 90.1% specificity, confirming its robust generalizability.

The generalizability of the prediction model was rigorously assessed in a well-characterized external validation cohort comprising 394 patients (Non-MASLD = 212, MASLD = 182). In this independent cohort, the combined model (TyG-BMI + CTI + SII) maintained excellent diagnostic performance, achieving an AUC of 0.940 (95% CI: 0.918–0.962), with a sensitivity of 84.6% and a specificity of 90.1% (Table 7 and Figure 2). Its discriminative power remained superior to that of any individual component. These results strongly affirm the model’s robustness and clinical transportability for MASLD risk stratification across diverse T2DM populations.

**Table 7:** Performance comparison between development and external validation cohorts.

Model	AUC (95% CI)	Sensitivity (%)	Specificity (%)	Youden’s Index
TyG-BMI	0.850 (0.813–0.887)	96.2	64.2	0.604
CTI	0.884 (0.853–0.916)	88.5	73.1	0.616
SII	0.801 (0.758–0.845)	76.4	75.0	0.514
TyG-BMI + CTI + SII	0.940 (0.918–0.962)	84.6	90.1	0.747

### 3.8 Validation with machine learning algorithms

**Figure 3:**
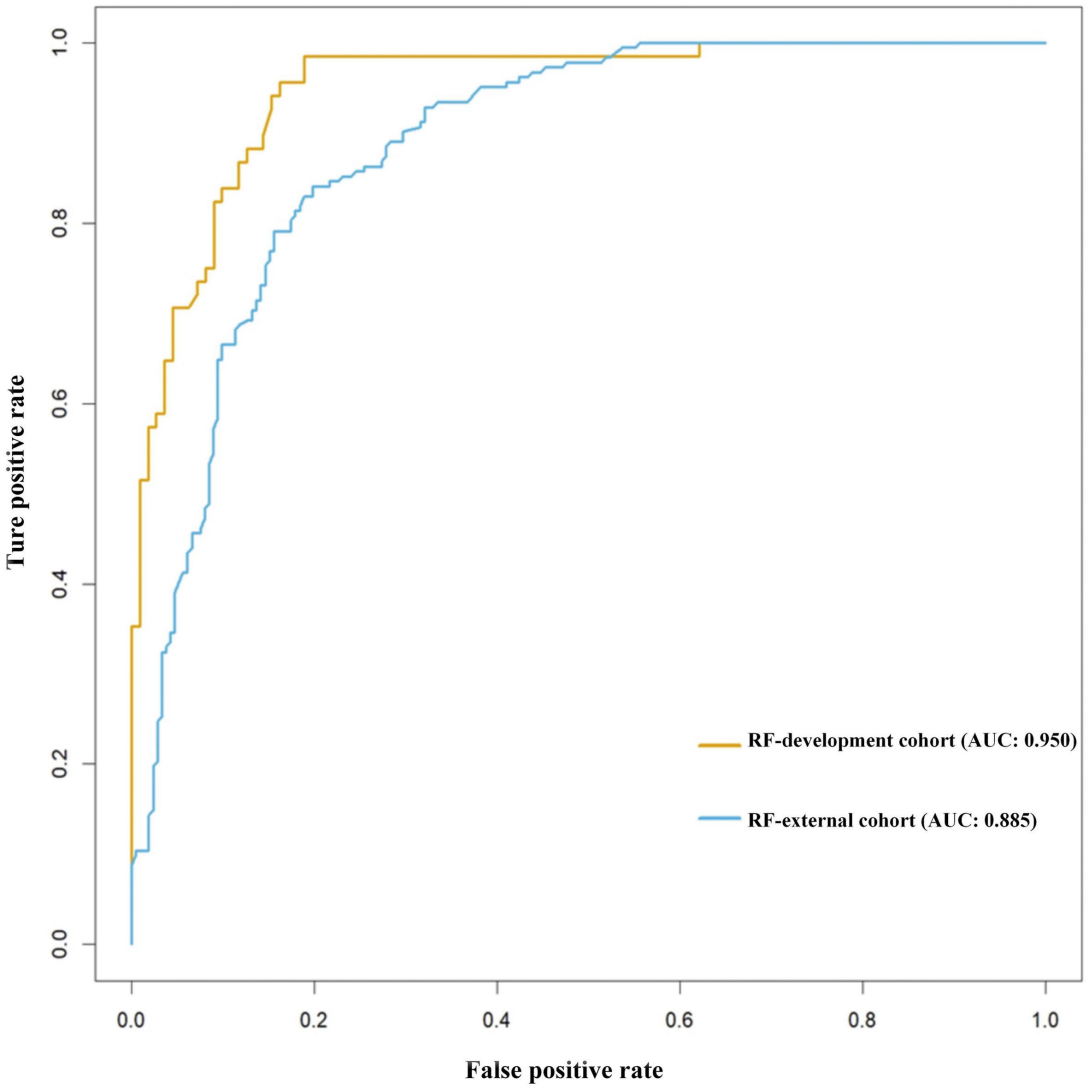
Receiver operating characteristic (ROC) curves of the random forest model for predicting MASLD in patients with T2DM. Receiver operating characteristic (ROC) curves illustrate the model’s discriminative ability in both the development (*n* = 796) and external validation (*n* = 394) cohorts. In the development cohort, the RF model integrating TyG-BMI, CTI, and SII achieved an AUC of 0.950 (recall 82.4%, precision 84.9%, specificity 91.0%). The model maintained good generalizability in the independent validation cohort, with an AUC of 0.885 (recall 81.3%, precision 79.1%, specificity 81.6%).

To further assess the robustness of the predictive value of TyG-BMI, CTI, and SII, and to explore potential nonlinear relationships, machine–learning–based validation was performed using a random forest (RF) algorithm. In the development cohort, the RF-full model demonstrated excellent discriminative performance, yielding an area under the receiver operating characteristic curve (AUC) of 0.950, with a recall of 82.4%, precision of 84.9%, specificity of 91.0%, accuracy of 87.7%, and F1 score of 0.836 (Table 8). This performance exceeded that of RF models built with any single indicator, confirming the synergistic contribution of metabolic and inflammatory information when the three indices were combined. The RF model retained good predictive ability in this external cohort, achieving an AUC of 0.885, recall of 81.3%, precision of 79.1%, specificity of 81.6%, accuracy of 81.5%, and F1 score of 0.802 indicating satisfactory generalizability across populations with different MASLD prevalence (Figure 3). Although modest attenuation in performance was observed compared with the development cohort, overall discrimination remained high, supporting the model’s stability.

**Table 8:** Predictive performance of random forest models using different feature combinations and external validation.

Model‌	Recall	Precision	Specificity	ROC	Accuracy	F1 score
RF-full model	0.824	0.849	0.910	0.950	0.877	0.836
RF-TYGBMI	0.735	0.833	0.910	0.929	0.844	0.781
RF-CTI	0.794	0.831	0.901	0.936	0.860	0.812
RF-SII	0.779	0.841	0.910	0.946	0.860	0.809
RF-external validation	0.813	0.791	0.816	0.885	0.815	0.802

Data are presented as recall, precision, specificity, area under the ROC curve (AUC), accuracy, and F1 score. RF-full model includes TyG-BMI, CTI, and SII. RF-TyG-BMI, RF-CTI, and RF-SII represent models constructed using a single predictor. RF-external validation was performed using an independent cohort.

## 4 Discussion

This study comprehensively evaluated the metabolic and inflammatory determinants of MASLD in patients with T2DM and established a combined predictive model based on TyG-BMI, CTI, and SII. In the present study, the prevalence of MASLD among patients with T2DM was 35.2%, slightly lower than figures reported in many population-based studies (24). First, diagnostic sensitivity is likely a major contributor: abdominal ultrasonography—used here—is less sensitive than MRI-PDFF for detecting mild steatosis and may underestimate true prevalence (25, 26). Secondly, the advanced mean age of our cohort may partly explain the observed prevalence. Age-related metabolic remodeling, together with frailty, involuntary weight loss, polypharmacy, and comorbidities, may alter hepatic fat accumulation in elderly patients with T2DM (27, 28). Third, in advanced liver disease (so-called “burnt-out” NASH) and in settings of severe malnutrition or marked catabolism, hepatic fat content may paradoxically decline (29, 30). Therefore, differences in imaging modality, cohort age, nutritional status, and strict exclusion criteria likely explain part of the discrepancy; notably, the external validation cohort exhibited a higher MASLD prevalence (46.2%), consistent with such sampling and diagnostic effects.

Beyond prevalence, our principal finding is that TyG-BMI, CTI, and SII are each significantly associated with MASLD and that their combination provided substantially greater discriminative ability than any single indicator. Collinearity assessment showed VIF values <2 (Supplementary Table 1), supporting the statistical validity of including all three indices together. Although TyG-BMI and CTI both incorporate triglyceride and glucose, their correlation coefficient was moderate (*r* = 0.62), and the combination captures complementary aspects: TyG-BMI emphasizes obesity-metabolic burden, while CTI integrates systemic inflammation via CRP. TyG-BMI, which combines an insulin-resistance surrogate (TyG) and anthropometric burden (BMI), was independently associated with MASLD after multivariate adjustment. This result is consistent with previous studies demonstrating that TyG-based indices are reliable markers of hepatic steatosis and metabolic syndrome (31–33). Mechanistically, TyG-BMI may reflect the dual burden of metabolic flux—characterized by hypertriglyceridemia and hyperglycemia—that promotes hepatic triglyceride accumulation, together with the contributory role of excess adiposity in facilitating lipid overflow into hepatocytes (34).

CTI demonstrated the largest effect size in our multivariate model. CTI combines CRP (inflammation) with glucose and triglycerides (metabolism). It may therefore capture the inflammatory-metabolic environment that drives liver fat accumulation and steatohepatitis progression (35). Our results accord with emerging literature highlighting inflammatory biomarkers as central mediators in MASLD pathogenesis and progression (36, 37). The stronger correlation of CTI compared with TyG-BMI alone underscores the added value of incorporating an inflammatory assessment.

SII, a composite index derived from neutrophil, lymphocyte, and platelet counts, was likewise an independent predictor and contributed materially to the combined model’s performance. Elevated SII likely reflects an activated innate immune response and relative lymphopenia, both of which have been implicated in metabolic inflammation and hepatic injury (38). Several recent clinical studies have reported associations between elevated SII (or related hematologic inflammation indices) and NAFLD severity and fibrosis risk, supporting its utility as an inexpensive systemic inflammation marker (39, 40).

The multivariable model also retained several conventional cardiometabolic factors as independent correlates of MASLD, including SBP, DBP, HDL-C, and LDL-C. Traditional metabolic and inflammatory markers (e.g., BMI, triglycerides, and hs-CRP) were not included simultaneously with TyG-BMI, CTI, or SII. This is because these composite indices already incorporate overlapping metabolic and inflammatory components. This approach was adopted to minimize redundancy and collinearity, and to allow more straightforward interpretation of the independent predictive value of the integrated indices. The association with DBP may reflect clustering of metabolic risk factors. Elevated diastolic pressure often co-occurs with insulin resistance and visceral adiposity, and may indicate vascular dysfunction contributing to hepatic microcirculatory stress (41, 42). It should be noted that systolic blood pressure and HDL-C were not independently associated in the adjusted model, which may be due to intercorrelations among cardiometabolic variables or insufficient variation within the study population.

The external validation cohort further supported the robustness and generalizability of the combined model across different clinical settings. This reproducibility suggests that the combined metabolic–inflammatory signature is not sample-specific. Instead, it may reflect fundamental pathophysiological processes applicable across different clinical settings.

In addition to conventional regression-based modeling, the robustness of the predictive value of TyG-BMI, CTI, and SII was further supported by machine–learning–based validation using a random forest algorithm. The RF model achieved excellent discrimination in the development cohort and maintained good performance in the external validation cohort, despite a modest attenuation in AUC. This decline is expected when models are applied to independent populations with different baseline characteristics and MASLD prevalence, suggesting limited overfitting rather than model instability. Significantly, the RF-full model consistently outperformed RF models based on single indicators, reinforcing the notion that integrating metabolic and inflammatory information provides complementary, non-redundant predictive value. Collectively, these findings indicate that the predictive utility of TyG-BMI, CTI, and SII is not confined to linear statistical assumptions but persists across different analytical frameworks.

Several limitations warrant consideration. First, the retrospective design, while providing substantial statistical power, inherently limits causal inference. Second, the use of ultrasonography rather than more sensitive imaging techniques (e.g., MRI-PDFF) may have led to under detection of mild steatosis, potentially introducing classification bias that could underestimate the true associations. Third, we did not assess liver fibrosis stage or distinguish between simple steatosis and steatohepatitis (NASH), as these require elastography or biopsy. Fourth, the single-center design may limit generalizability to other populations, although external validation from another institution partially mitigates this concern. Fifth, we lacked data on lifestyle factors and medication use, which could influence metabolic and inflammatory indices. Sixth, collinearity among indices, while statistically acceptable (VIF < 2), necessitates caution when interpreting individual coefficient signs.

Specific follow-up studies should include: (1) prospective multicenter cohorts using MRI-PDFF as the reference standard to validate the optimal cutoffs and assess the model’s predictive performance for incident MASLD; (2) investigation of longitudinal changes in TyG-BMI, CTI, and SII in relation to MASLD regression or progression following lifestyle or pharmacological interventions; (3) cost-effectiveness analyses to determine whether routine calculation of these indices in T2DM patients is economically justified in primary care settings; (4) integration of these indices with fibrosis markers to improve risk stratification for advanced liver disease.

## 5 Conclusion

TyG-BMI, CTI, and SII are independently and synergistically associated with MASLD in patients with T2DM. The combined model exhibits excellent diagnostic performance and external validity, outperforming individual markers. This laboratory-based composite index provides a practical and noninvasive approach for early detection and risk stratification of MASLD, supporting its potential use in routine diabetes management.

## Data Availability

The datasets generated and analyzed during the current study are not publicly available due to restrictions in the ethical approval concerning patient confidentiality but are available from the corresponding author on reasonable request. Requestors will need to sign a data access agreement.

## Supplementary material

SUPPLEMENTARY FIGURE 1 Boxplots of TyG-BMI, CTI, and SII between MASLD and non-MASLD groups in T2DM patients. Boxplots show the distribution of TyG-BMI, CTI, and SII in patients with MASLD (*n* = 280) and without MASLD (*n* = 516). The lower and upper boundaries of each box represent the first and third quartiles, respectively; the horizontal line inside each box indicates the median; whiskers extend to 1.5 times the interquartile range (IQR); and circles denote outliers. TyG-BMI, triglyceride‑glucose‑body mass index; CTI, C‑reactive protein‑triglyceride‑glucose index; SII, systemic immune‑inflammation index; MASLD, metabolic dysfunction‑associated steatotic liver disease; T2DM, type 2 diabetes mellitus.
