# Supplementary figures and images for: Predictive value of TyG-BMI, CTI, and SII in identifying metabolic dysfunction-associated steatotic liver disease among patients with type 2 diabetes mellitus
Source: Front Nutr. 2026 May 8;13:1808180. doi: 10.3389/fnut.2026.1808180 (PMC13194478; doi:10.3389/fnut.2026.1808180)

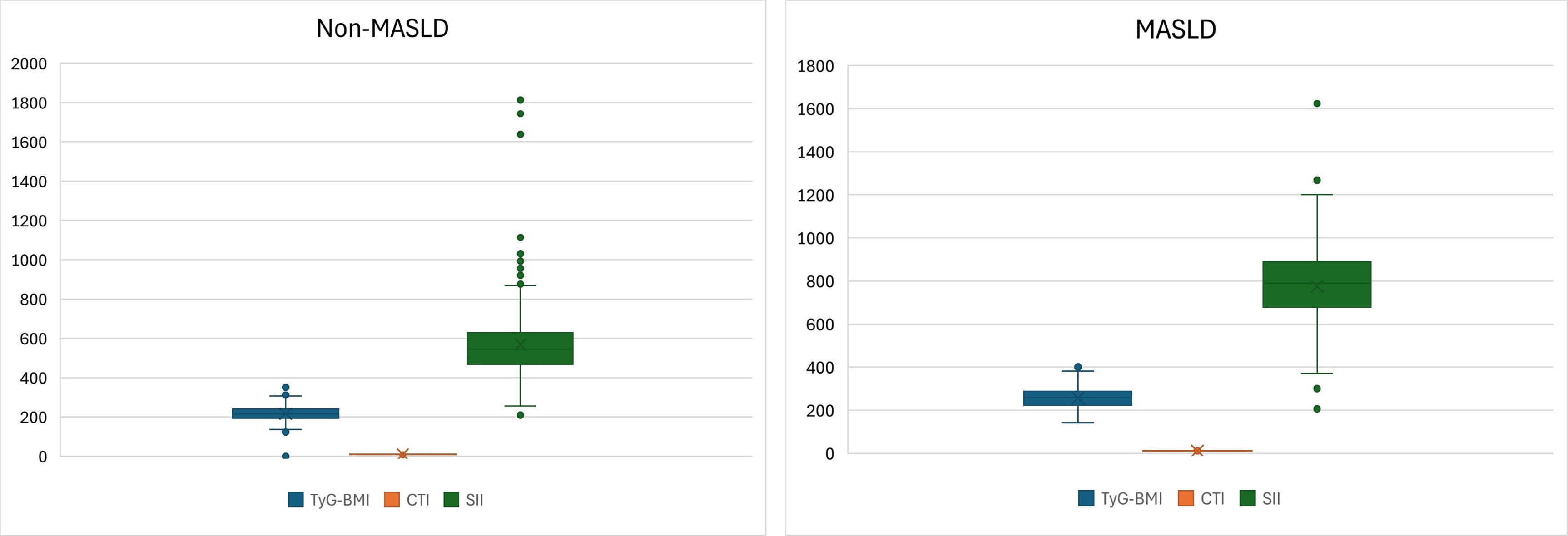

Supplement: SUPPLEMENTARY FIGURE 1 — Boxplots of TyG-BMI, CTI, and SII between MASLD and non-MASLD groups in T2DM patients. Boxplots show the distribution of TyG-BMI, CTI, and SII in patients with MASLD (n = 280) and without MASLD (n = 516). The lower and upper boundaries of each box represent the first and third quartiles, respectively; the horizontal line inside each box indicates the median; whiskers extend to 1.5 times the interquartile range (IQR); and circles denote outliers. TyG-BMI, triglyceride‑glucose‑body mass index; CTI, C‑reactive protein‑triglyceride‑glucose index; SII, systemic immune‑inflammation index; MASLD, metabolic dysfunction‑associated steatotic liver disease; T2DM, type 2 diabetes mellitus. [file Image_1.JPEG]
