# Supplementary material for: Predictive value of TyG-BMI, CTI, and SII in identifying metabolic dysfunction-associated steatotic liver disease among patients with type 2 diabetes mellitus
Source: Front Nutr. 2026 May 8;13:1808180. doi: 10.3389/fnut.2026.1808180 (PMC13194478; doi:10.3389/fnut.2026.1808180)
Supplement: Supplementary file 2 [file Table_1.DOCX]

**Supplementary Table 1. Correlation matrix and variance inflation factors (VIF) for TyG-BMI, CTI, and SII**

| Variable | TyG-BMI | CTI | SII | VIF |
| --- | --- | --- | --- | --- |
| TyG-BMI | 1.00 | 0.62 | 0.48 | 1.82 |
| CTI | 0.62 | 1.00 | 0.55 | 1.76 |
| SII | 0.48 | 0.55 | 1.00 | 1.45 |

**Supplementary Table 2. Comparative performance of different machine learning classifiers in the development cohort**

|  | Recall | Precision | Specificity | ROC | Accuracy | F1 score |
| --- | --- | --- | --- | --- | --- | --- |
| KNN | 0.853 | 0.841 | 0.901 | 0.886 | 0.827 | 0.770 |
| SVM | 0.765 | 0.776 | 0.865 | 0.927 | 0.866 | 0.829 |
| RF | 0.853 | 0.806 | 0.874 | 0.950 | 0.877 | 0.836 |
| GBM | 0.824 | 0.849 | 0.910 | 0.947 | 0.860 | 0.815 |
| XGBOOST | 0.809 | 0.821 | 0.892 | 0.946 | 0.860 | 0.820 |

**Supplementary Table 3. Pairwise comparison of ROC curves using DeLong test**

| Model 1 | Model 2 | ΔAUC | 95% CI | Z value | *P* value |
| --- | --- | --- | --- | --- | --- |
| TyG-BMI + CTI + SII | TyG-BMI | 0.1512 | 0.117–0.185 | 8.673 | <0.001 |
| TyG-BMI + CTI + SII | CTI | 0.0844 | 0.061–0.108 | 7.118 | <0.001 |
| TyG-BMI + CTI + SII | SII | 0.0787 | 0.054–0.103 | 6.364 | <0.001 |
| TyG-BMI | CTI | 0.0668 | 0.026–0.108 | 3.212 | 0.001 |
| TyG-BMI | SII | 0.0725 | 0.024–0.121 | 2.922 | 0.004 |
| CTI | SII | 0.0058 | -0.035–0.046 | 0.277 | 0.782 |
